# Challenges associate with microbiome diversity, glucocorticoids, and condition in a wild songbird

**DOI:** 10.1038/s41598-026-42507-x

**Published:** 2026-03-13

**Authors:** Morgan C. Slevin, Jennifer L. Houtz, Maren N. Vitousek, Rindy C. Anderson

**Affiliations:** 1 Department of Biological Sciences, Florida Atlantic University, Davie, FL USA; 2 Department of Ecology and Evolutionary Biology, Cornell University, Ithaca, NY USA; 3 Department of Biology, Allegheny College, Meadville, PA USA; 4 Cornell Lab of Ornithology, Ithaca, NY USA

**Keywords:** Ecology, Ecology, Microbiology, Zoology

## Abstract

Wild animals experience daily fitness challenges, and the resulting stress responses can disrupt an animal’s gut microbiome. Given the links between health and microbiome composition, it is essential to understand how challenges affect microbial communities. We showed that a fitness challenge significantly alters the cloacal microbiome of free-living Northern cardinals (*Cardinalis cardinalis*), and these shifts covary with changes to the glucocorticoid stress response, condition, and beak ornamentation. Treatment significantly related to beta diversity, while it only influenced alpha diversity indirectly through interactions with health-related traits. Birds held for an extended time before release showed greater cloacal microbiome community changes 11 days later vs. birds administered a simulated territorial intrusion challenge, or control birds administered no challenge. We also detected relationships between beta diversity and change in body condition and beak ornamentation. Birds showing the greatest alpha diversity decrease and largest beta diversity between timepoints experienced the greatest corticosterone response to handling. Finally, several Amplicon Sequence Variants were differentially abundant in challenged birds compared to control birds. To our knowledge, this study is the first to demonstrate proximate effects of fitness challenges on the microbiome of an adult, free-living songbird, while simultaneously tracking changes in glucocorticoid levels, body condition, and ornamentation—providing a uniquely integrative perspective on how stress shapes host-microbe interactions in the wild.

**Supplementary Information:**

The online version contains supplementary material available at 10.1038/s41598-026-42507-x.

## Introduction

Multicellular organisms are host to many microbiomes: the symbiotic and pathogenic microbiota that naturally persist on or within tissues and organs. Microbiota can influence multiple aspects of host health, including immune function^1^, body condition^2^, and the stress response^3^; these host changes, in turn, can further shape microbiomes^4^. As a result, external threats to host communities can directly impact host fitness and may also indirectly affect fitness by altering microbiomes^5^. A microbiome with lower diversity can leading to immunodeficiency or decreased nutrient assimilation^6^ and higher pathogen relative abundances^4^. Conversely, microbiomes with high diversity may be more resistant to pathogen invasion and more resilient following disturbance^7^, however the benefits of high alpha diversity are not universal across taxa^8^.

During the hormonal stress response, a common disturbance to an organism’s homeostatic state, a hormonal cascade connecting an animal’s hypothalamus, pituitary, and adrenal glands (the hypothalamic-pituitary-adrenal or HPA axis) elevates the circulating concentration of glucocorticoid hormones. Corticosterone is the primary glucocorticoid in birds, and while some birds also produce cortisol at lower levels, in those that do, it appears to be most relevant in immune tissues and during development, while corticosterone is most common in the plasma of adult birds^9^. Increases in circulating corticosterone can shift behavior and physiology in ways that reduce the effect of a stressor on the individual^10^. However, chronically elevated corticosterone often has injurious side effects on a host’s microbiome. For example, corticosterone can activate an immune response^11,12^, decreasing bacterial species richness. Stress can also alter foraging behavior^13^, further impacting host physiology. While the relationships between stressors, corticosterone, and the microbiome are well established in some mammals and in poultry^14^, forming the microbiota-gut-brain axis, relatively little is known about these connections in wild birds. Also, the microbiota-gut-brain axis is bidirectional (stress affects the microbiome and vice versa), but most studies have only artificially manipulated corticosterone levels in wild or captive birds to investigate downstream microbiome effects^12^. Therefore, testing the effect of natural stressors on the gut microbiome is still needed.

Common methods to induce stress in avian subjects include capture and restraint and simulated territorial intrusions (STIs). Human capture and restraint mimic a predation event and reliably induce an acute stress response^15^. This method allows researchers to estimate an individual’s baseline corticosterone prior to capture (if the first sample is collected within 3 min of capture), as well as the speed of corticosterone change and the scope (size) of that change in response to the stress of capture and subsequent restraint^16^. A STI of a male into a conspecific male’s territory using audio playback is also widely used to elicit a natural aggressive response in songbirds. In many species, the defending male perceives the intrusion as an immediate threat, causing an acute stress response and release of corticosterone^17^. Stress response severity can depend on the duration and intensity of the STI. For example, Northern cardinals (*Cardinalis cardinalis*) exposed to 60 min of STI had greater baseline corticosterone compared to cardinals exposed to < 5 min^18^.

**Fig. 1:**
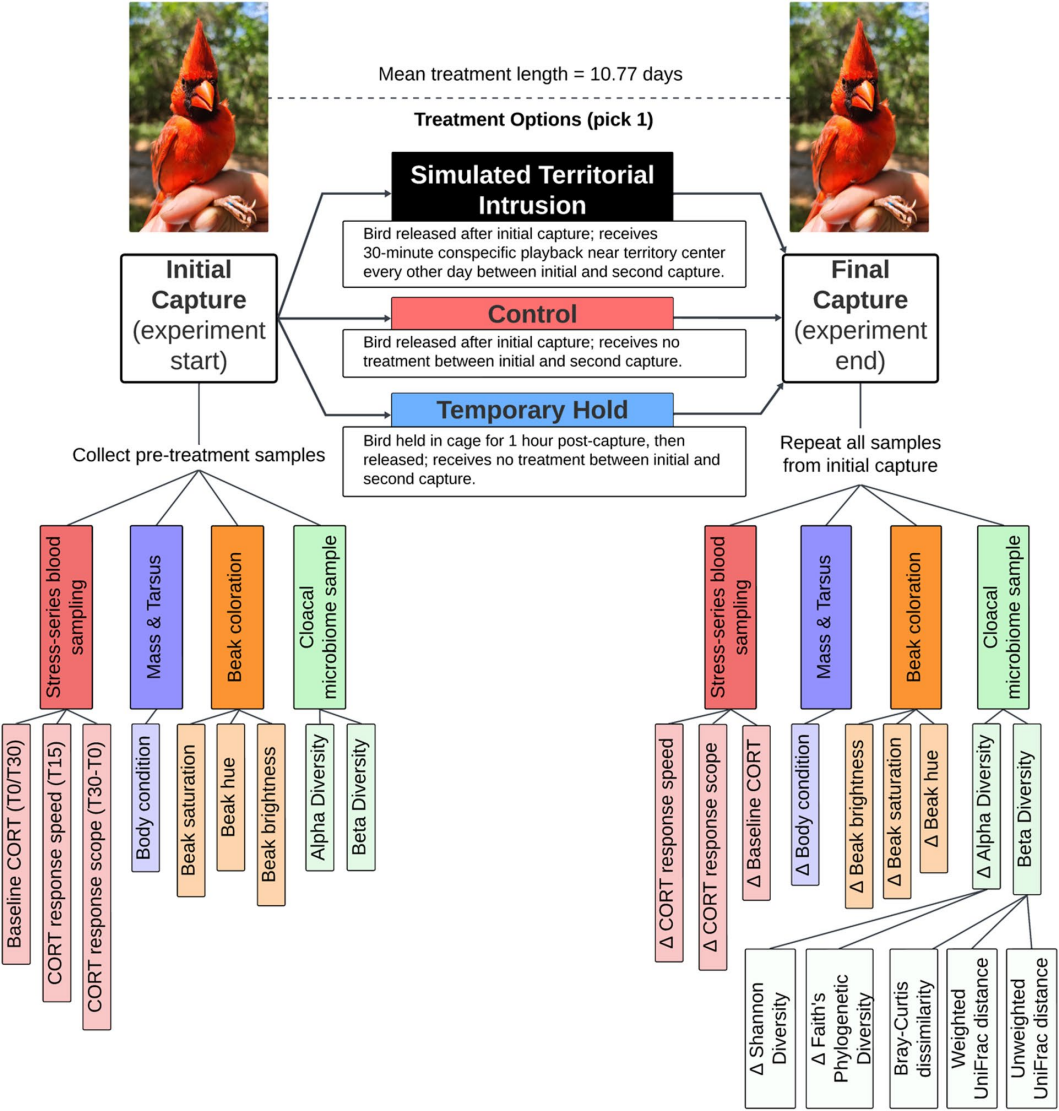
A concept map of the experimental design. Male Northern cardinals were captured for baseline pre-treatment samples, randomly assigned one of three treatments, and then recaptured 1–2 weeks later for post-treatment re-sampling. All samples are repeated measures paired by bird and nested within treatment group, where the “∆” symbol signifies the change in each variable between pre-treatment and post-treatment sampling and “CORT” stands for corticosterone.

Songbirds are an excellent study system for investigating the gut-stress relationship because most species show rapid, consistent neuroendocrine responses to stressors and social interactions^19^. To improve our understanding of how a bird’s gut microbiome responds to stress in the wild, we conducted an experiment with free-living Northern cardinals, a common territorial songbird. We tested how gut microbial diversity responded to one of three treatments administered between pre- and post-treatment captures separated by approximately 11 days: (a) STI, (b) Temporary Hold, or (c) no treatment as the control (Fig. 1). The Temporary Hold treatment (extended restraint time after pre-treatment capture) applies a single, brief challenge, while the STI treatment is a stressor that is repeated over multiple days. Therefore, this design also allows us to compare chronic and acute stressors. We assessed three dynamic measures of health: corticosterone, body condition, and beak coloration. Body condition (mass relative to body size) is often used as an indicator of fitness. Tissue ornament coloration that is dependent on carotenoid pigmentation, such as the red-orange beak of cardinals, is thought to be an honest signal of health because the pigment cannot be synthesized internally by birds and must be obtained through diet^20^. A bird’s beak is vascularized tissue, offering dynamic health and immunocompetence estimates^21,22^, and it is a sexually selected signal in Northern cardinals^23^. A previous study within this cardinal population revealed males with higher alpha diversity in their gut microbiota had better body condition and differed in their beak ornamentation, but not in corticosterone concentration^24^. Therefore, measuring body condition and beak coloration as added proxies for changes in health, in addition to glucocorticoids, may add to our understanding of how these various challenges affect the gut microbiome.

We hypothesized that stress causes a significant shift in the cloacal microbiome in male Northern cardinals and predicted that it would alter the microbiome community structure, increase circulating corticosterone concentration, and reduce alpha diversity, beak color ornamentation, and body condition. We predicted the STI and Temporary Hold groups would experience greater changes in the above measures compared to the control group. These results offer insights into how wild populations respond to stressors like urbanization and have practical implications for captive breeding programs and wildlife rehabilitation centers, where stress commonly alters behavior and impairs appetite, healing, and overall treatment success^25^.

## Results

Overall, our treatments impacted cardinals’ cloacal microbiome beta diversity (dissimilarity between pre- and post-treatment samples’ taxonomic diversity and Amplicon Sequence Variant (ASV) relative abundances), but not alpha diversity (log ratio of pre- and post-treatment samples: $\mathrm{log}\frac{\mathrm{p}\mathrm{o}\mathrm{s}\mathrm{t}}{\mathrm{p}\mathrm{r}\mathrm{e}}$). Males in the STI and Temporary Hold groups showed larger beta diversity between sampling timepoints than Control males. Although treatment did not directly impact alpha diversity, alpha diversity fluctuations correlated with birds’ changes in their fitness-associated traits: beak coloration, corticosterone response, and body condition.

### Treatment and alpha diversity

**Fig. 2:**
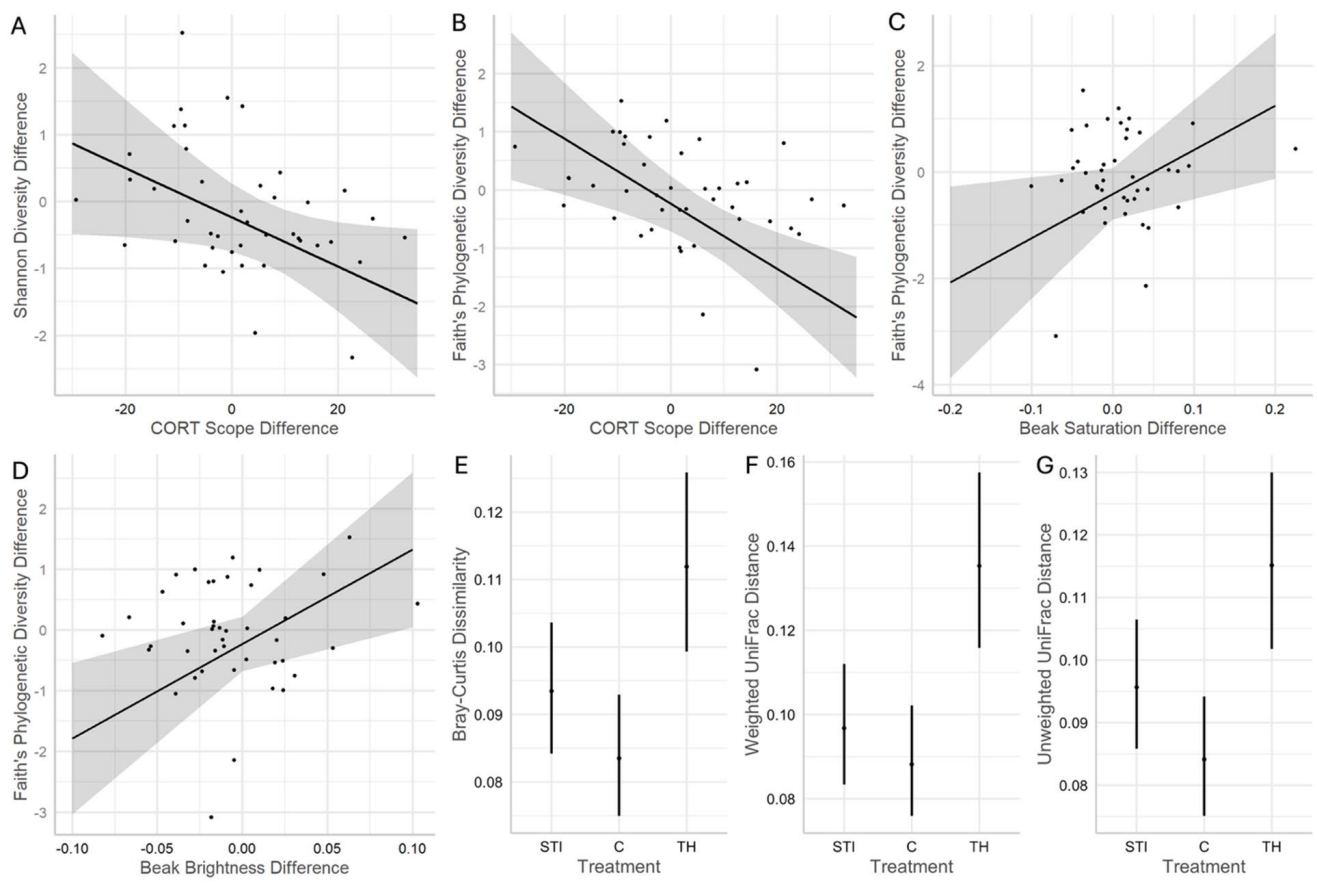
Predicted data and trendlines for change in Shannon and change in Faith’s Phylogenetic Diversity and change in predictor values between pre- and post-treatment microbiome sampling of Northern cardinal males. Change in corticosterone (CORT) scope significantly predicted change in (**A**) Shannon Diversity and (**B**) Faith’s Phylogenetic Diversity, and Faith’s Phylogenetic Diversity was significantly predicted by change in (**C**) beak saturation and (**D**) beak brightness. (** E**–**G**) Beta diversity between a bird’s sample timepoints differed among treatment groups (STI=Simulated Territorial Intrusion, C=Control, TH=Temporary Hold).

Shannon Diversity (SD) change negatively related to corticosterone scope change; i.e., more Shannon Diversity was lost when a bird’s corticosterone response to the stress of capture restraint increased between samples (*z*=-2.05, *p* = 0.040, Fig. 2). Additionally, Shannon Diversity change showed weak interactions between treatment and beak brightness change (*p* = 0.060, Figure S1A), baseline corticosterone change (*p* = 0.099, Figure S1B), and corticosterone speed change (*p* = 0.086, Figure S1C), but none were significant after multiple test correction. Faith’s Phylogenetic Diversity (FPD) change significantly related to corticosterone scope change (diversity decreased when corticosterone response size increased; *z*=-3.32, *p* < 0.001), beak saturation change (diversity decreased if beak saturation decreased; *z* = 2.13, *p* = 0.033), and beak brightness change (diversity decreased if brightness decreased; *z* = 2.59, *p* = 0.010; Fig. 2). FPD change also showed a significant interaction between treatment and beak saturation change (*p* = 0.034), and a nearly significant interaction between treatment and beak brightness change (*p* = 0.058), but neither remained significant after multiple test correction.

### Treatment and beta diversity

Beta diversity (change in taxonomic diversity and relative abundance between timepoints) significantly differed among treatments. Overall, beta diversity was largest for Temporary Hold birds, moderate for STI birds, and lowest for Control birds (Bray-Curtis: χ2 = 12.32, *p* = 0.002; non-significant after multiple test correction; Unweighted UniFrac: χ2 = 18.47, *p* < 0.001; and Weighted UniFrac: χ2 = 10.10, *p* = 0.006; Fig. 2). For Unweighted UniFrac, community dissimilarity between timepoints in Temporary Hold birds was 137% larger than in Control birds (adjusted *p* = 0.002) and 120% larger than in STI birds (adjusted *p* = 0.047), with STI beta diversity values being 113% higher than in Control birds (adjusted *p* = 0.071; Fig. 2F, Supplementary Information Table S2).

### Body condition’s response to treatment

**Fig. 3:**
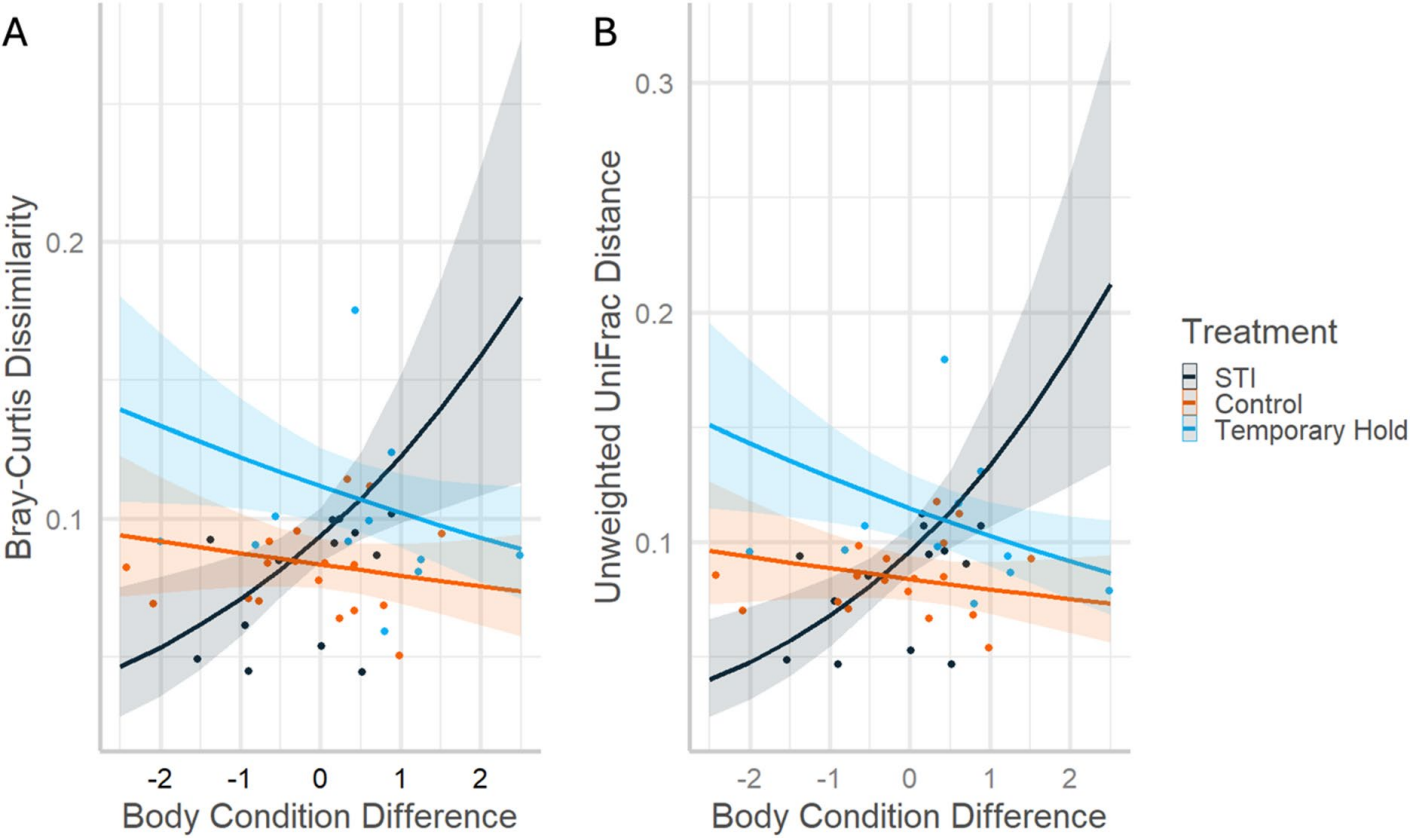
Predicted data and trendlines for the significant interaction between treatment and change in body condition, explaining variation in beta diversity dissimilarities (**A**: Bray-Curtis Dissimilarity; **B**: Unweighted UniFrac Distance) between timepoints of Northern cardinal microbiome samples. “STI” stands for simulated territorial intrusion.

The amount that birds’ fitness-related traits changed between timepoints depended on the treatment received. First, body condition change significantly predicted Bray-Curtis (χ2 = 12.49, *p* = 0.002) and Unweighted UniFrac (χ2 = 17.87, *p* = 0.001) but only for STI birds, where community shifts were greatest when birds gained the most mass between samples (Fig. 3A). STI birds with the largest body condition increase had 244% and 202% larger Bray-Curtis distance than Control and Temporary Hold birds, respectively, while STI birds with the largest body condition decrease had 51% and 68% lower Bray-Curtis distance than Control and Temporary Hold birds, respectively. This trend and the associated relative effect sizes were similar for Unweighted UniFrac (Fig. 3B, Supplementary Information Table S3).

### Beak coloration’s response to treatment

**Fig. 4:**
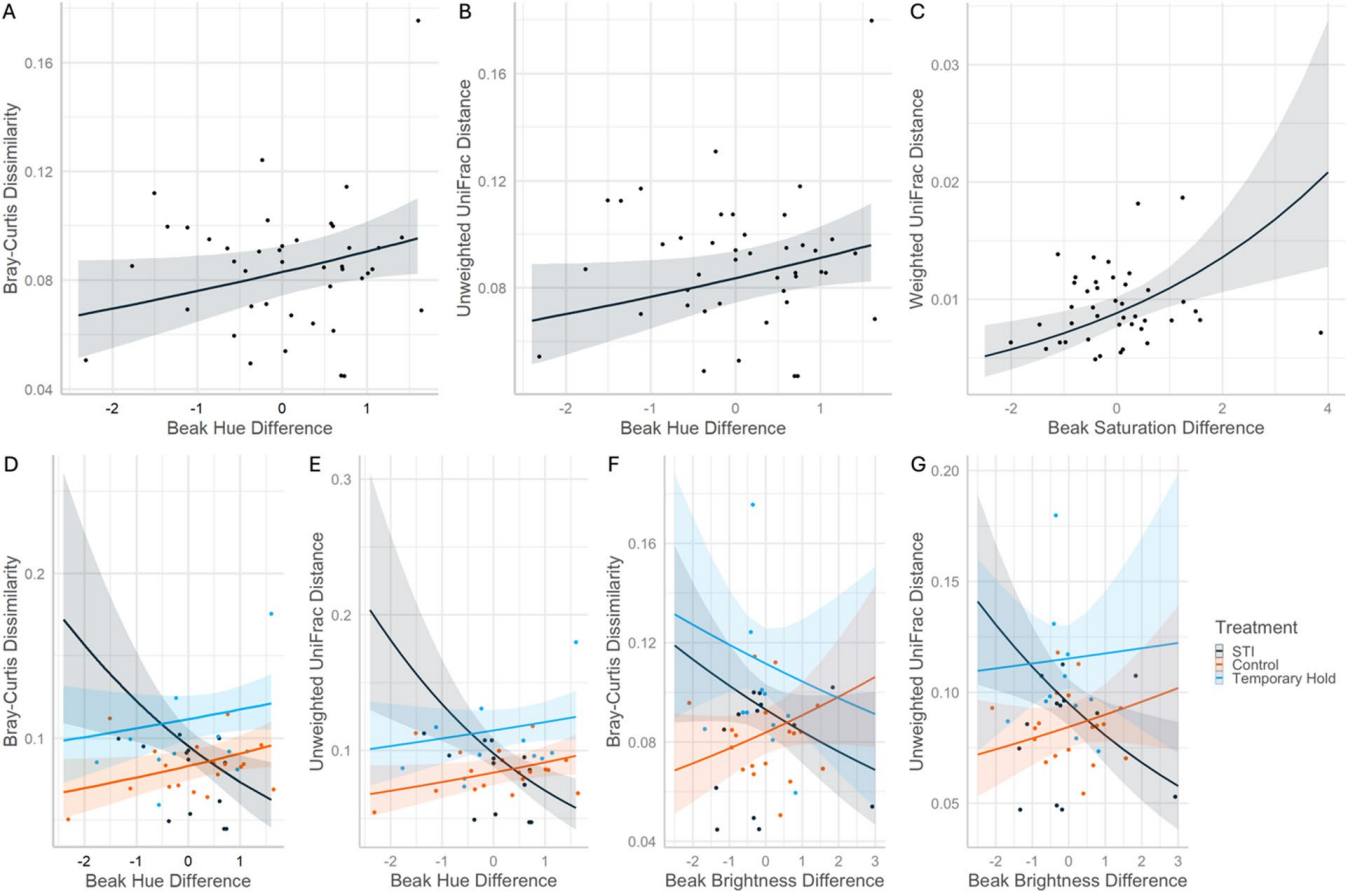
Predicted data and trendlines for the significant relationships between beta diversity dissimilarities between timepoints of Northern cardinal microbiome samples and change in beak hue (**A**,**B**,**D**,**E**), brightness (**F**,**G**), and saturation (**C**). “STI” stands for simulated territorial intrusion.

A bird’s change in beak coloration between captures also related to beta diversity. We noted significant relationships with change in beak hue (Bray-Curtis: *z* = 2.01, *p* = 0.044; Unweighted UniFrac: *z* = 1.93, *p* = 0.053), and interaction effects between treatment and changes in hue, brightness, and saturation. The beak hue of STI birds was significantly more likely to become more orange when community change was largest (Fig. 4A and B). When STI birds’ beaks showed the greatest shifts towards red hues, Bray-Curtis dissimilarity was 35% and 48% lower than Control and Temporary Hold groups, respectively, but 171% and 90% greater than Control and Temporary Hold birds when hue showed the greatest shifts towards orange (Fig. 4D). A similar interaction effect was seen for Unweighted UniFrac distance (Fig. 4E, Supplementary Information Table S4).

Beak brightness changes significantly negatively related to beta diversity for STI birds (Unweighted UniFrac distance; Fig. 4G, Supplementary Information Table S4), but not Control or Temporary Hold birds. STI birds with the largest brightness increases had 41% and 51% lower Unweighted UniFrac distances (i.e., less community change) than Control and Temporary Hold birds, respectively, while STI birds with the largest brightness decreases had 199% and 130% greater Unweighted UniFrac distances than Control and Temporary Hold birds. Beak brightness also significantly interacted with treatment to influence Bray-Curtis dissimilarity; however, this relationship was not significant after multiple test correction (Fig. 4F, Supplementary Information Table S4). Finally, beak saturation change negatively related to Weighted UniFrac (*z* = 3.14, *p* = 0.002); birds whose saturation increased the most also experienced the greatest microbiome community shifts between samples (Fig. 4C).

### Corticosterone’s response to treatment

**Fig. 5:**
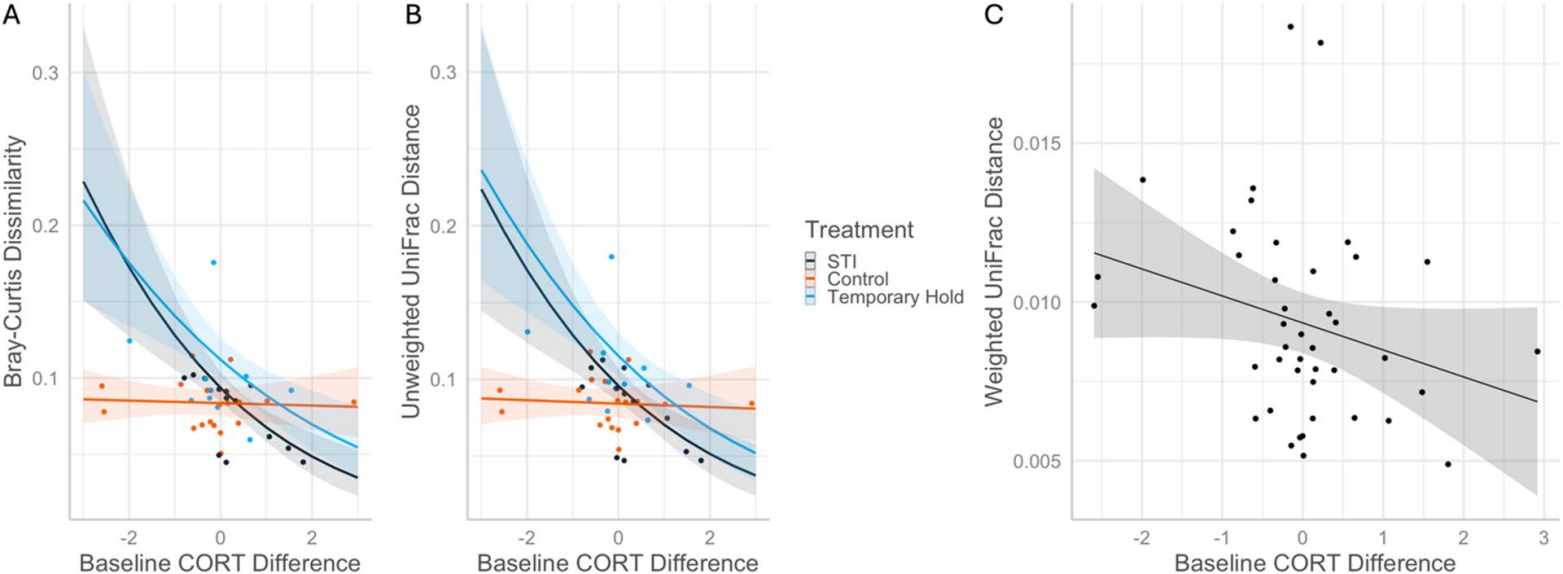
Predicted data and trendlines of beta diversity between timepoints of Northern cardinal microbiome samples, as predicted by a significant interaction effect between treatment and change in baseline corticosterone (**A**,**B**) and a significant relationship between change in baseline corticosterone and Weighted UniFrac distance (**C**). “STI” stands for simulated territorial intrusion and “CORT” for corticosterone.

In general, cardinal males in the STI and Temporary Hold groups, but not Control males, showed significant positive relationships between beta diversity and both variations in baseline corticosterone and the size of the corticosterone response. When baseline corticosterone showed the greatest decreases between timepoints, STI and Temporary Hold birds’ Bray-Curtis dissimilarities were 266% and 251% greater than that of Control birds, respectively, whereas they were 52% and 34% smaller than that of Control birds when baseline corticosterone showed the greatest increases (Bray-Curtis: χ2 = 19.73, *p* < 0.001; Unweighted UniFrac: χ2 = 18.04, *p* < 0.001; Fig. 5). Weighted UniFrac distance was significantly negatively related to change in baseline corticosterone (*z*=-2.42, *p* = 0.016, Fig. 5C) with no interaction between timepoints (i.e., Weighted UniFrac distance decreased with increasing baseline corticosterone difference for all treatments; *z* = 3.00, *p* = 0.003).

**Fig. 6:**
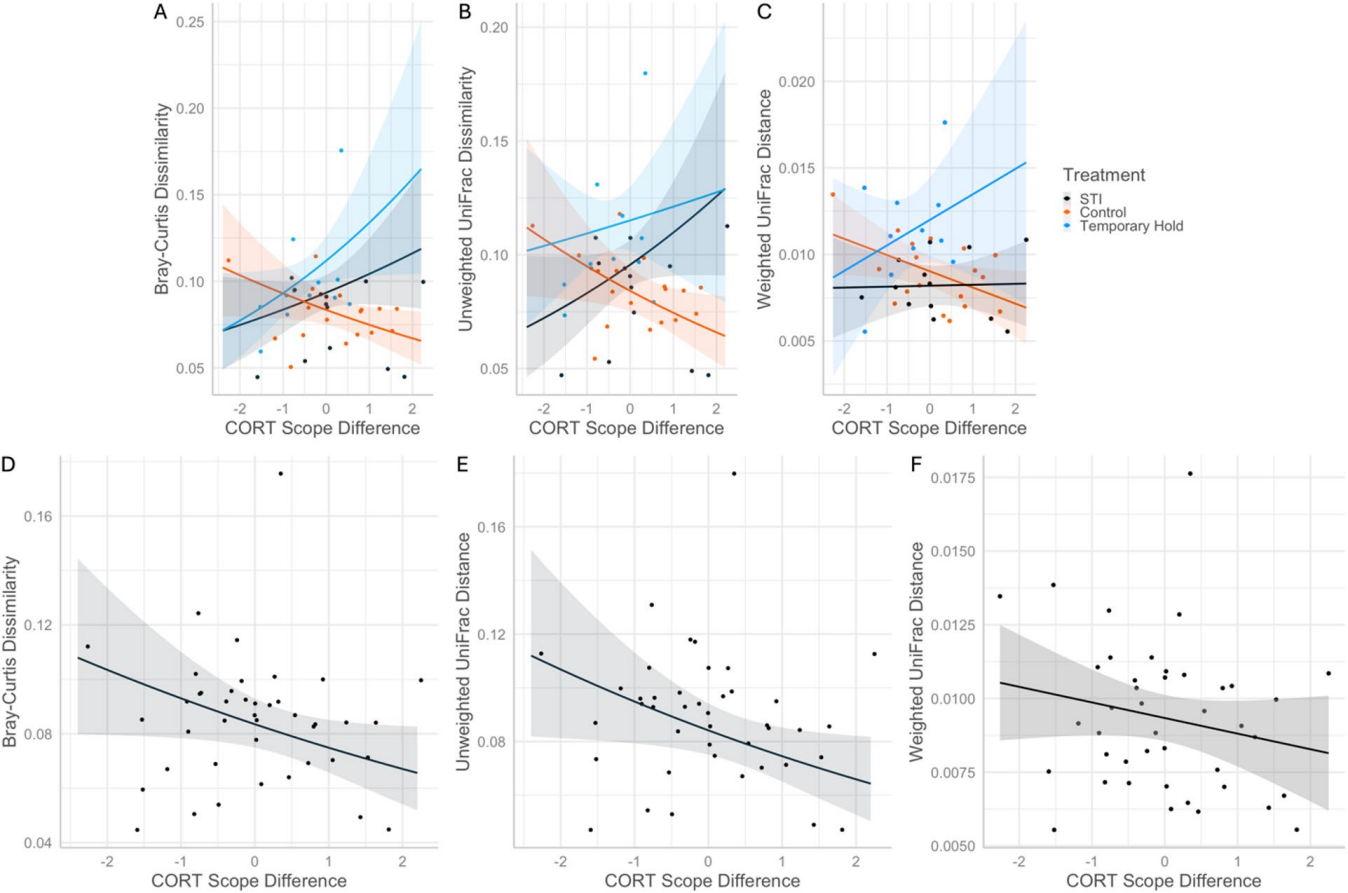
Predicted data and trendlines modeling beta diversity between timepoints of Northern cardinal microbiome samples. There was a significant interaction effect between treatment and change in corticosterone (CORT) scope (**A**–**C**), and a significant relationship between change in CORT scope and all beta diversity metrics (**D**–**F**). “STI” stands for simulated territorial intrusion.

Corticosterone scope fluctuations also showed a significant interaction effect with treatment for all beta diversity measures (Bray-Curtis: χ2 = 1.22, *p* = 0.004, Unweighted UniFrac: χ2 = 9.30, *p* = 0.010, Weighted UniFrac: χ2 = 7.27, *p* = 0.026). While Control birds showed a weak negative relationship, STI and Temporary Hold birds showed a positive relationship between the two (except STI birds’ Weighted UniFrac distance, which showed no relationship; Fig. 6, Supplementary Information Table S5). The change in corticosterone scope also showed significant negative relationships directly with Bray-Curtis dissimilarity (*z*=-1.99, *p* = 0.046, Fig. 6) and Unweighted UniFrac distance (*z=*-2.14, *p* = 0.032, Fig. 6) independent of an interaction, and a relationship approaching significance with Weighted UniFrac distance (*z=*-1.82, *p* = 0.069, Fig. 6), but these relationships are largely uninformative given the opposite slopes among groups due to the interaction effect. Finally, corticosterone speed change significantly related to Weighted UniFrac such that one increased with the other (z = 0.26, *p* = 0.003).

### Treatment’s effect on abundances of specific ASVs

Depending on the treatment group, several ASVs were differentially abundant in post-treatment cloacal microbiome samples. Compared to the Control group, males in the Temporary Hold group had significantly greater abundances of the *Staphylococcus* genus (coefficient = 4.1, *p* < 0.001; Supplementary Information Figure S4), and males in the STI group showed significantly greater abundances of the genera *Devosia* (coefficient = 3.7, *p* < 0.001) and *Cellulomonas* (coefficient = 3.1, *p* < 0.001; Supplementary Information Figure S4). Additionally, ∆baseline corticosterone concentration associated positively with relative abundance of the *Mycoplasma* genus (*p* < 0.001), and negatively with the genera *Bacillus* (*p* < 0.001), *Nocardioides* (*p* = 0.004), *Chlamydiales* (*p* = 0.003), *Microvirga* (*p* = 0.003), and the species *Methylobacterium komagatae* (*p* = 0.003; Supplementary Information, Figure S5). Finally, 45 ASVs positively correlated and one negatively correlated with SD change (Supplementary Information Table S5).

## Discussion

We tested the hypothesis that stress causes alterations in the bacterial cloacal microbiome of a free-living songbird, the Northern cardinal. We predicted the stressful effects of capture would indirectly alter the cloacal microbiome, then tested whether a subsequent challenge induces a more pronounced shift than capture alone, and whether different types of challenges vary in their impact on the microbiome. We found partial support for our hypothesis that a post-capture challenge can impact the microbiome, and we learned that an extended restraint after capture tended to have a larger impact on this change than repeated simulated territorial intrusions (Fig. 2E-G). However, this was only true for beta diversity between pre- and post-treatment sample pairs, whereas alpha diversity was only indirectly impacted by treatment type through interactions with fitness-associated variables. Finally, we identified specific ASVs that were more enriched in fitness-challenged birds than in control birds, and a small number of ASVs whose abundances correlated with baseline corticosterone change. This experiment adds to the growing body of microbiome studies aimed at elucidating the interactions between a wild vertebrate’s cloacal microbiome and its fitness.

Beginning our discussion with the treatment’s impact on the cloacal microbiome, we found that beta diversity changed more between timepoints if we held the bird for an additional 60 min post-capture (Temporary Hold treatment). Because the Control group’s mean beta diversity was > 0, our capturing protocol alone may be enough to alter the microbiome (Fig. 2E-G), but this effect is likely idiosyncratic. Given that this capture event simulates predation, we are unsurprised that lengthening this event for the animal exacerbated the effects on the microbiome. Weighted and Unweighted UniFrac distances differed more between treatment groups than Bray-Curtis dissimilarity, suggesting that the microbiome samples differed between timepoints based on phylogeny and relative abundances more than ASV identities.

The effect of treatment we measured supports the hypothesis that capture stress alters the microbiome of a wild songbird. Activation of the HPA axis can trigger macrophage concentration increases when the immune system triggers during stress, which can attack the host’s bacteria^26^. Further support for this treatment effect comes from the fact that birds in the Temporary Hold and the STI groups had the greatest rise in corticosterone scope between sample timepoints when their cloacal microbiome community showed the greatest differences (i.e., beta diversity; Fig. 6). Crucially, Control birds did not show this relationship. Also, with respect to the above trend, birds in the STI group had smaller effect sizes than Temporary Hold males. These relationships imply that temporary confinement induced a larger effect of the stress response on the microbiome than repeated territory intrusions. Avian ecologists widely use STIs to study territorial defense and endocrine responses, or to capture birds^27^. For example, repeated STIs impacted behavioral response in the black redstart (*Phoenicurus ochruros*), but not its corticosterone^28^. Similarly, in a previous Northern cardinal study, corticosterone response scope was unaffected by STI duration^29^, but baseline corticosterone levels were higher in males exposed to 60 min of playback vs. those given < 5 min of playback, confirming that STIs do induce a stress response in this species. Our results support downstream effects from this in that beta diversity levels of STI-group birds were between those of Control and Temporary Hold birds (Fig. 2E-G).

Our alpha diversity analysis supports the above results; cardinals that experienced the greatest increases in corticosterone scope exhibited the greatest alpha diversity decreases (Fig. 2). This was true for both Shannon and Faith’s Phylogenetic Diversity indices, providing additional confidence that the stress of capture changed the microbiome, at least in part, through HPA axis activation. Likewise, in one of the first studies to describe such a proximate interaction in a free-living vertebrate, Stothart et al.^30^ showed that the Shannon diversity of wild red squirrels’ (*Tamiasciurus hudsonicus*) oral microbiomes decreased after capture, and their fecal glucocorticoid levels increased. This mirrors the relationship between corticosterone scope and Faith’s Phylogenetic Diversity that we found.

In contrast to the above results, we found the largest baseline corticosterone increases between sample points in males with the least amount of microbiome differences between samples, and only in STI and Temporary Hold birds (Fig. 5). There is no consensus on what constitutes an adaptive response to stress in a microbiome. Microbial communities may gain stability when the host increases corticosterone in response to capture, a response that makes evolutionary sense because elevated glucocorticoids function to protect the organism from anything harmful. Or, perhaps, microbiome community shifts in the face of stress may simply be a healthy response to challenge. Direct corticosterone manipulations in a study with repeated-measures microbiome sampling are necessary to elucidate the proximate connection between glucocorticoids and the gut microbiome. Defining microbiome eubiosis versus dysbiosis is still in its infancy due to limitations in functional data^31^, therefore future research to answer this question needs to involve intervention study designs.

Birds with the greatest shifts in beak hue also had the greatest beta diversity between timepoints, supporting our hypothesis that health challenges can change the microbiome. This is only partially supported because we saw this for STI birds but not Temporary Hold or Control birds (Fig. 4). We also noted a significant positive trend between beak saturation change and Faith’s Phylogenetic Diversity regardless of treatment group (Fig. 2), suggesting that birds who experienced a health challenge dramatic enough to reduce beak saturation also experienced phylogenetic diversity loss in their cloacal microbiomes. This supports honest signaling theory, that the beak honestly and dynamically signals Northern cardinal health, and that our treatment duration and severity were enough to induce and detect beak coloration changes. Although prior research provides mixed results on how carotenoid-based coloration responds to stress^22,32^, few studies have explored the gut microbiome’s potential role connecting glucocorticoids to coloration. However, recent research shows correlations between ornamentation and the microbiome in the cardinal^24^, and a growing body of research supports an ornamentation-microbiota relationship^33,34^.

Despite the above relationships, microbiome changes only related to body condition through a treatment interaction (Fig. 3). We predicted that birds exhibiting the greatest microbiome shifts would show declines in body condition, but we only found mixed support for this. Birds in the Control and Temporary Hold groups that lost the most mass between captures displayed the greatest changes to their microbiome, while the opposite was true for STI birds. Body condition appeared to covary with differences in ASV presence or composition, rather than shifts in relative abundance, because the above was only seen through Unweighted UniFrac and Bray-Curtis metrics. We made our prediction above because HPA axis activation can cause body mass reductions by consuming energy reserves to cope with stress. Zebra finches (*Taeniopygia guttata*) stressed through capture and handling had significant mass reductions over 4 weeks^21^, with other studies noting a similar phenomenon^13,35^. Therefore, we were not surprised when a post-hoc generalized linear model showed that cardinals in our study with the greatest body condition declines across the treatment had the greatest increases in corticosterone scope (z=-2.8, *p* = 0.005) and baseline corticosterone (z=-1.8, *p* = 0.073; Supplementary Information Figure S3). However, recent research challenges the traditional hypothesis that stress reduces body mass through mobilizing fat reserves^36^. Instead, the mass reductions we measured may be through a different mechanism that the glucocorticoid response mediates, such as stress-induced changes in foraging behavior^13^.

Finally, we identified multiple ASVs in post-treatment samples that significantly correlated with baseline corticosterone change regardless of treatment group. Most notable among these was *Bacillus*, whose relative abundance inversely related to baseline corticosterone change. In addition to their popularity as a probiotic for humans, many *Bacillus* strains are studied in poultry research for their antimicrobial properties. For example, “*B. subtilis* PB6,” a strain isolated from healthy chickens’ guts, has shown antimicrobial activity against common avian gut pathogens like *Clostridium* sp., *Escherichia coli*, and *Campylobacter* sp.^37^, reduced disease symptoms^38^, outperformed standard antibiotics in measures of host health^39^, and even promotes the growth of beneficial microbes like *Lactobacillus* and *Bifidobacterium* sp.^37^. While we recognize our sequencing depth only revealed the *Bacillus* ASV to the genus level, and there are many species of *Bacillus*, *B. subtilis* is not the only beneficial species described in the avian gut. Coccidiosis-challenged chickens fed ten different *Bacillus* types, compared to birds not fed *Bacillus*, had less weight loss, reduced lesion scores, and lower oocyst counts. A few *Bacillus* strains even showed improved gene expression of inflammatory response proteins and antioxidants^40^. Despite these accounts showing that *Bacillus* sp. may be beneficial features in an avian gut, we must conservatively conclude that changes in this ASVs simply show a compositional shift; additional studies are needed to test for a functional significance to the ASV.

Three other ASVs correlated with baseline corticosterone change. *Mycoplasma* increased in relative abundance with increasing baseline corticosterone, while *Microvirga* and *Nocardioides* decreased in relative abundance with increasing baseline corticosterone. All three genera include common avian pathogens; however, it remains unclear whether HPA axis activation increases the relative abundance of pathogens or instead reduces susceptibility to opportunistic infections^12^. Additionally, we could not identify the specific strains present; some of these genera, such as *Mycoplasma*, include numerous non-pathogenic strains that are part of the normal gut flora in wild birds^41^. As a result, it is difficult to determine the direction or significance of their associations with changes in baseline corticosterone; nonetheless, we report these findings to guide future research with their potential relevance.

Several ASVs were differentially enriched among treatment groups. Temporary Hold birds’ cloacal microbiomes showed >4x greater *Staphylococcus* than Control birds, and STI birds had >3x *Devosia* and *Cellulomonas* than Control birds. As with many genera, the *Staphylococcus* genus is quite varied, including common pathogens such as *S. aureus*, while other strains are commensals or can even display inhibitory activity against common avian pathogens like *Enterococcus faecalis* and *E. coli*^42^. Likewise, *Devosia* is largely undescribed in avian gut research, with one study testing the effects of varying isoleucine feed levels on broiler chickens. The group that received the highest dose grew the slowest, and *Devosia* was one of the genera enriched in this group^43^. While human medical research finds *Devosia* may be a signal of gut disease in patients, such as colorectal cancer^44^, changes in *Devosia* abundance may only reflect compositional changes to avian cloacal microbiomes, much like those reported for *Bacillus* and others above.

### Conclusions and future implications

The treatment effects on cloacal microbiome beta diversity and its interaction with corticosterone scope offer insight into how natural challenges, such as social stress from a simulated territorial intrusion (STI), differ from unnatural challenges like brief captivity in shaping microbial communities. Our results indicate that the unnatural challenge of captivity caused greater disruption to homeostasis than the natural stressor, as evidenced by greater compositional shifts in the microbiome, elevations in stress-induced corticosterone, and body mass reduction in Temporary Hold birds. Stress-challenged birds showed enrichment of potentially pathogenic genera such as *Staphylococcus*, while individuals with greater increases in baseline corticosterone were more likely to exhibit depletion of *Bacillus*, a typically beneficial genus. Interpreting the microbiome effects of captivity across studies remains challenging due to the variability of host species, captivity duration, and treatment protocols. For example, wild animal microbiome researchers tend to capture individuals for repeated-measure sampling in a highly controlled laboratory space, but individuals are typically not returned to the wild due to either regulations against reintroduction, sacrificial sampling, or future research projects. While in captivity, various treatments may be given in attempts to replicate wild conditions^45^ or to introduce additional stress challenges^5^. Although these studies are a crucial first step to understanding wild animal microbiomes, sampling animals in a natural setting should produce more ecologically relevant results. While our findings demonstrate that even brief captivity can alter the microbiome, the specific proximate effects of captivity on microbial communities remain poorly understood in wild songbirds.

This study provides some of the first empirical evidence linking a fitness challenge to microbiome shifts in an adult, free-living songbird, with concurrent changes in glucocorticoid levels, body condition, and ornamentation—offering a rare integrative view of host physiology, condition, and microbial dynamics in the wild. Taken together, our findings underscore the importance of incorporating microbiome dynamics into studies of ecological stress and life history, offering new insights into how environmental challenges may influence host fitness through physiological and microbial pathways.

## Methods

### Experimental design and capture sampling

We used a between-groups, within-subjects experimental design during the Northern cardinal breeding seasons of 2021 and 2022 (February-July) at Tree Tops Park, Davie, Florida, USA. We randomly assigned each male (identified by plumage patterns) to a treatment group after initial capture and sampling (Fig. 1).

For the “STI” treatment group, we released each male on its territory after sampling. Beginning the day after capture and continuing until the following capture, we administered a repeated STI treatment. Across treatment groups, the average treatment length was 11 days, with a range of 2–7 STIs received. We ran each STI for 30 min, repeated every other day until recapture (e.g., if a bird was recaptured after 7 days, it received 3 STIs, but if it eluded recapture until 13 days, it received 6 STIs). To guard against the fact that some birds were more difficult to recapture than others, we repeated STIs until capture to avoid unintended potential recovery from a challenge that ended before a bird was successfully recaptured. We approached each male’s territory from a different direction for each STI and mounted a speaker playing male Northern cardinal song, placed in a unique location each time ≥ 50 m from a known territory border and 1–2 m above the ground, hidden in vegetation. We created playback files from recordings of our study population of male Northern cardinals, and tracks were played at ≥ 80 dB (we confirmed amplitude at 1 m using a B&K Precision 732 A sound level meter, A-weighting). To minimize habituation to the treatment, we used a unique stimulus each time for a male, we never used a familiar male’s songs in a recording (current or former neighbor), and we ran each STI at a random time between sunrise and 1100. It is unknown if the number of STIs impacted reported outcomes, however we controlled for this potentially confounding factor by incorporating treatment length into our models (see *Statistical analysis*).

Each bird from “Temporary Hold” birds, our other treatment group, was housed alone in a metal cage with two wooden perches (56 cm wide x 38 cm tall x 31 cm deep) at its capture location for 1 h after sampling. To avoid inadvertent microbiome changes during the hold, we did not provide food, only a sterilized dish with bottled spring water. We covered the cage with a breathable cloth and placed it in the shade until release onto the bird’s territory. We sanitized cages with a 10% bleach solution spray between captures.

The third and final group, “Control” birds, were released after sampling and left undisturbed until recapture, avoiding their territories between captures. All treatments lasted an average of 11 days (range = 5–19 d), then we collected the same samples as during the initial (pre-treatment) capture. Birds were tracked and recaptured using unique color band combinations attached during the first capture. We released all males on their territory after collecting samples from the second capture. See “Ethics statement and methodological reports” section below for details of ethical animal handling and experimental design.

To capture and sample cardinals, we used established methods that have been validated in a previous study of the same population^24^. We used a digital scale and digital calipers to measure mass and tarsus, respectively, to index body condition $\left(\frac{mass}{tarsus}\right)$. We attached a metal United States Geological Survey band after capturing each bird using a mist net, and a unique combination of three plastic color bands to identify individuals without recapture. We also collected a series of three blood samples to observe the hormonal response of corticosterone to the stress of capture and handling. The first sample, collected within 3 min of capture, provides an estimate of baseline corticosterone; the second sample, collected 15 min later, allows us to assess the speed of corticosterone increase; the third sample, collected 30 min after the first sample, shows the scope of how much corticosterone increased from baseline levels^46^. Each bird sat quietly in a cloth bag between samples, and this method has been well-validated in avian ecology^16^ and with this study population^24^. We collected a maximum of 75 µl blood per sample via brachial venipuncture into microhematocrit capillary tubes (Fisherbrand, Catalog No. 22-362-566). Blood was transported on ice (≤ 4 h) until plasma decantation in the laboratory by centrifugation for 5 min at 12,000 rpm using a McKesson Microhematocrit Centrifuge. Plasma was stored at -80 °C until corticosterone quantification using an Arbor Assays Enzyme Immunoassay kit (Catalog No. K014-H; cross reactivities listed in Supplementary Information Table S9). No hemolysis was detected across plasma samples.

Finally, we quantified beak ornamentation using established methods^24,47^ to collect coloration data from standardized photographs. We photographed each male Northern cardinal in profile with a digital Canon SLR camera. We extracted coloration metrics (hue, saturation, and brightness) associated with carotenoid concentration^48^ from a quantitative color pattern analysis framework (QCPA) run in the micatoolbox plugin (version 2)^49^ for ImageJ. This framework uses multispectral images, which we created from our original beak photographs using a cone catch model built from the blue tit (*Cyanistes caeruleus*) model of the avian visual system and a color standard (X-Rite ColorChecker Passport) in the background of every photograph. QCPA condenses each beak into clusters of similar colors so we may extract data from each cluster. Our value for beak brightness (perceived light reflectance) came from the double wavelength cone catch quanta value, while saturation’s value (color patch purity) came from channel stimulation relative to the achromatic center. We quantified hue (the color the beak appears to be) in the R package pavo2^50^ from the short, medium, and long wavelength values, calculating the angle of the vector between the color and its origin in a triangular RGB colorspace.

### Microbiome sample preparation, sequencing, and data processing

To sample the cloacal microbiome, we used validated methods from our previous work in this system^24,51^. We swabbed each bird’s cloaca with a sterilely-packaged swab (Puritan 25-3316-U 6′′) and used sterilized scissors to clip the tip into an autoclaved 2-ml centrifuge tube containing 1 ml sterile RNAprotect (Qiagen). We then transported it on ice for storage in the laboratory at -80 °C for later DNA extraction using a Qiagen PowerSoil DNA Extraction Pro kit (following manufacturer instructions modified for swab samples). We also extracted 1 negative control sample (1 sterile swab in 1 ml RNAprotect in a microcentrifuge tube) for every 12 cloacal swab samples for a total of 8 negative controls. We verified quality using a Nanodrop 2000 (A260/A280 ratio 1.7-2.0, concentration ≥ 5 ng/µl). We followed the Earth Microbiome Protocol for PCR in triplicate^52^ and used modified primers 515 F/806R with Illumina adaptors to amplify the V4 region of the 16S rRNA gene. Cornell’s Biotechnology Resource Center processed final pooled PCR products for quantification, normalization, library preparation, and sequencing (Illumina MiSeq paired-end 2 × 250 bp). We sequenced 86 total microbiome samples from 42 unique individuals, with 8 negative controls. We split samples across two sequencing runs based on the year they were collected. We sequenced 34 samples in the first batch collected 28 May – 17 July 2021, with 3 negative controls, and 52 microbiome samples in the second batch collected 8 February – 19 May 2022, with 5 negative controls.

We performed initial processing of raw sequences using Quantitative Insights into Microbial Ecology 2 (QIIME2, version 2019.7)^53^, trimming sequences of their primers to join them for denoising and quality filtering per nucleotide^54–57^. We used the Scikit-learn system and the SILVA 132 database to annotate each Amplicon Sequence Variant (ASV)^58,59^, then removed all chloroplast, mitochondria, and unassigned sequences. We built a midpoint-rooted phylogenetic tree using FASTTREE^60^ by aligning ASVs using MAFFT^61^ and then masking them^62^. Raw sequences and other project data are accessible through Dryad (10.5061/dryad.41ns1rnnq), while the snakemake^63^ pre-configured coding loop files used in QIIME2 are available on GitHub^64^. Finally, we used the decontam package^65^ in R version 4.3.3^66^ to decontaminate samples, with DNA yield and prevalence in negative controls as input conditions with a threshold of 0.05 for significance testing. 31 ASVs were identified as contaminants and removed prior to analysis (Supplementary Information Table S8). Mean sequencing depth was 14758.34 ± 631.9927 reads before decontamination and 15552.34 ± 647.417 afterwards.

We estimated alpha diversity in R through Shannon and Faith’s Diversity (phyloseq package’s estimate_richness function^67^ and the picante package’s pd function^68^, respectively). We determined how much each bird’s alpha diversity changed between pre- and post-treatment samples by calculating each sample pair’s log ratio of diversity values ($\mathrm{log}\frac{\mathrm{p}\mathrm{o}\mathrm{s}\mathrm{t}}{\mathrm{p}\mathrm{r}\mathrm{e}}$); an increase in diversity returned a positive value while negative values indicated a diversity decrease^69^. We built beta diversity dissimilarity matrices (phyloseq’s distance function) for the metrics Bray-Curtis, Weighted UniFrac, and Unweighted UniFrac. Each matrix value, between 0 and 1, compared two samples for similarity, with 1 being the least similar. From each matrix, we extracted the beta diversity dissimilarity value comparing a bird’s pre- and post-treatment samples as our measure of the difference between a bird’s pair of samples. For all other variables, we report the difference in that measure between a birds’ two timepoints.

### Statistical analysis

To test for an effect of treatment on the change in the cloacal microbiome between sample timepoints, we built linear models with microbiome diversity as the response and treatment group as a predictor variable (Table 1). To assess alpha diversity, we built one model for change in Shannon Diversity and one for change in Faith’s Phylogenetic Diversity. We built one model for each of three beta diversity metrics: Bray-Curtis, Weighted UniFrac, and Unweighted UniFrac. Because we predicted that the fitness-related traits measured during capture may influence change in diversity between timepoints, we included interaction effects between treatment and change in body condition, beak coloration (brightness, saturation, and hue), and glucocorticoids (baseline corticosterone, corticosterone response speed, and corticosterone response scope). We also included sequencing batch as a predictor to assess any impact of the sequencing run on the change in diversity. A random effect of bird ID was not needed because we calculated all values as the difference between each bird’s paired samples. We used the glmmTMB function^70^ in R to fit all models, with a beta distribution for beta diversity models and a Gaussian distribution for alpha diversity models. We strived to catch each bird approximately 8–11 days after initial capture for a standardized treatment length, but sometimes this was not possible due to field logistics and difficulty recapturing some males. Because treatment length significantly related to all three beta diversity metrics or had a significant interaction with treatment, we controlled for this variation by dividing each bird’s beta diversity value by the number of days between samples. A relationship did not exist between treatment length and either alpha diversity metric, so we did not include this measure of control in the alpha diversity models.

**Table 1:** Final models used for alpha and beta diversity analyses. An * indicates an interaction effect between the parameter before the * and everything in parentheses afterwards. All predictor variables (except Treatment and batch) represent a change in value between pre-treatment and post-treatment and are denoted with a “∆” to indicate this difference, calculated as post-treatment value minus pre-treatment value. “CORT” stands for “corticosterone.” All samples are paired by bird through their inherent calculation methods and therefore without the need for a random effect of bird ID.

Response	Predictors	Error family
$\mathrm{log}\frac{ShannonDiversityposttreament}{ShannonDiversitypretreament}$	Treatment * (∆body condition + ∆beak brightness + ∆beak saturation + ∆beak hue + ∆baseline CORT + ∆CORT speed + ∆CORT scope) + batch	Gaussian
$\mathrm{log}\frac{Fait{h}^{{\prime}}sPhylogeneticDiversityposttreament}{Fait{h}^{{\prime}}sPhylogeneticDiversitypretreament}$	Treatment * (∆body condition + ∆beak brightness + ∆beak saturation + ∆beak hue + ∆baseline CORT + ∆CORT speed + ∆CORT scope) + batch	Gaussian
$\frac{Bray-Curtisbetweentimepoints}{treatmentlength\left(days\right)}$	Treatment * (∆body condition + ∆beak brightness + ∆beak saturation + ∆beak hue + ∆baseline CORT + ∆CORT speed + ∆CORT scope)	Beta
$\frac{UnweightedUniFracbetweentimepoints}{treatmentlength\left(days\right)}$	Treatment * (∆body condition + ∆beak brightness + ∆beak saturation + ∆beak hue + ∆baseline CORT + ∆CORT speed + ∆CORT scope)	Beta
$\frac{\mathrm{W}\mathrm{e}\mathrm{i}\mathrm{g}\mathrm{h}\mathrm{t}\mathrm{e}\mathrm{d}\mathrm{U}\mathrm{n}\mathrm{i}\mathrm{F}\mathrm{r}\mathrm{a}\mathrm{c}\mathrm{b}\mathrm{e}\mathrm{t}\mathrm{w}\mathrm{e}\mathrm{e}\mathrm{n}\mathrm{t}\mathrm{i}\mathrm{m}\mathrm{e}\mathrm{p}\mathrm{o}\mathrm{i}\mathrm{n}\mathrm{t}\mathrm{s}}{treatmentlength\left(days\right)}$	Treatment * (∆body condition + ∆beak brightness + ∆beak saturation + ∆beak hue + ∆baseline CORT + ∆CORT speed + ∆CORT scope)	Beta

We centered and scaled all variables prior to modeling, checked all models for collinearity (check_collinearity function in the performance package^71^, and inspected residuals (simulateResiduals function in the DHARMa package^72^. We used anovas (Anova function (type = 3) in the car package^73)^ to test the effect of each predictor and treatment interaction on change in diversity. We inspected directionality and effect sizes (ggeffects package^74^ for all predictors and interactions that were significant (*p* < 0.05) or approaching significance (0.05 < *p* < 0.10) and conducted post-hoc pairwise comparisons when treatment or an interaction was significant (test_predictions function, using the Benjamini-Hochberg method for multiple test corrections).

Finally, we conducted post-hoc differential abundance analysis using the MaAsLin2 package^75^. We tested males’ post-treatment cloacal microbiome samples for associations between treatment group and relative abundances of specific ASVs, as well as any multivariate associations between fitness-related traits and differential abundances. We used the default settings of log-transformation and total sum scaling normalization, and corrected significance values using the Benjamini-Hochberg false discovery method.

### Ethics statement and methodological rdesign comparing samples from individual male Northerneports

All animals were handled following an approved protocol with Florida Atlantic University’s Institutionalstudy design comparing samples from individual Animal Care and Use Committee, and all methods are carried out in accordance with relevant guidelines and regulations. Methods were conducted in accordance with the ARRIVE guidelines for in vivo experiments, reviewed briefly as follows. (1) *Study design*: We used a three-group (two treatments and one control), two-sample study design comparing samples from individual male Northern cardinals’ first and second samples (Fig. 1). (2) *Sample size*: 42 male cardinals were captured and sampled twice. 11 males were in the Temporary Hold group, 14 males were in the STI group, and 18 were in the Control group (1 male was captured in the first and second sample year and therefore placed in different treatment groups to avoid pseudo-replication: Control 1st year and STI 2nd year). Sample size was not decided a priori but was by necessity small because it is difficult to capture songbirds more than once in such a short period of time; a maximum sample target was set to 20 birds per group. (3) *Inclusion and exclusion criteria*: Birds were only used if they exhibited normal behavior before and after release from the first sampling capture; birds’ samples were excluded if one or both microbiome samples did not pass rarefaction. (4) *Randomisation*: Males were randomly assigned to treatment group with a random number generator using Microsoft Excel. Neighboring males never received treatment during the same period to avoid confounding effects. (5) *Blinding/Masking*: MCS was aware of group allocation during the data collection, but not during data processing or analysis, as random sample IDs were assigned for the latter stages. (6) *Outcome measures*: The primary outcome measure was diversity changes between first and second sampling points; other outcome measures compared between sampling timepoints include the birds’ corticosterone response to stress, beak coloration, and body condition. (7) *Statistical methods*: We built linear models in Program R with diversity as the response (1 model for each metric) and predictor variables of treatment and its interaction with corticosterone response to stress, beak coloration metrics, and body condition. We included sequencing batch to account for any confounding batch effects, and all models were checked for validity and multicollinearity; we report effect sizes and significance of a predictor’s effect on change in diversity. (8) *Experimental animals*: We only included free-living Northern cardinals (*Cardinalis cardinalis*) that were putatively adult (after-hatch-year) male (based on plumage and behavior). Some males had been captured in previous years as part of an associated study^24^. (9) *Experimental procedures*: Each subject was captured and released twice over the study using mist nets and audio lures to collect cloacal microbiome samples, beak photographs, body morphometrics, and three blood samples. Captures were separated by an average of 11 days, with a treatment (or lack thereof for the control group) occurring between captures. (10) *Results*: Summary-by-group statistics for outcome measures can be found in Supplementary Information Tables S6 and S7, and effect sizes for each relationship are in Tables S2-S4.

## Supplementary Information

Below is the link to the electronic supplementary material.

Supplementary Material 1

## Data Availability

Data are available from the Dryad Digital Repository: https://doi.org/10.5061/dryad.41ns1rnnq.
