# Supplementary material for: Challenges associate with microbiome diversity, glucocorticoids, and condition in a wild songbird
Source: Sci Rep. 2026 Mar 13;16:8511. doi: 10.1038/s41598-026-42507-x (PMC12988219; doi:10.1038/s41598-026-42507-x)
Supplement: Supplementary file 1 — Supplementary Material 1 [file 41598_2026_42507_MOESM1_ESM.docx]

SUPPLEMENTARY INFORMATION

Challenges associate with microbiome diversity, glucocorticoids, and condition in a wild songbird

Morgan C. Slevin^1*^, Jennifer L. Houtz^2,3^, Maren N. Vitousek^2,4^, Rindy C. Anderson^1^

^1^Florida Atlantic University, Department of Biological Sciences, Davie, Florida, USA

^2^Cornell University, Department of Ecology and Evolutionary Biology, Ithaca, New York, USA

^3^Allegheny College, Department of Biology, Meadville, Pennsylvania, USA

^4^Cornell Lab of Ornithology, Ithaca, New York, USA

^*^corresponding author: [slemc425@gmail.com](mailto:slemc425@gmail.com)

TABLES

Table S1. Pairwise comparisons and relative effect sizes between treatment groups with respect to beta diversity between a Northern cardinal male’s pair of samples pre- and post-treatment. STI=Simulated Territorial Intrusion, TH=Temporary Hold, and C=Control.

| Parameter | Beta Diversity Metric | Pairwise Comparison | Means  Contrast | Adjusted *P* |
| --- | --- | --- | --- | --- |
| Treatment | Bray-Curtis | TH – C | 0.04 | 0.231 |
|  |  | TH – STI | 0.04 | 0.736 |
|  |  | STI – C | 0.00 | 0.231 |
|  | Unweighted UniFrac | TH – C | 0.03 | 0.002 |
|  |  | TH – STI | 0.02 | 0.047 |
|  |  | STI – C | 0.01 | 0.071 |
|  | Weighted UniFrac | TH – C | <0.01 | 0.001 |
|  |  | TH – STI | <0.01 | 0.005 |
|  |  | STI – C | <0.01 | 0.334 |

Table S2. Pairwise comparisons and relative effect sizes for the significant interaction effect between ∆body condition and beta diversity between Northern cardinal microbiome sample timepoints, organized by treatment and beta diversity metric. STI=Simulated Territorial Intrusion, TH=Temporary Hold, and C=Control. Effect Size “Low” and “High” refer to, respectively, the effect size at the lowest and highest end of the spectrum of values for ∆body condition.

| Beta Diversity Metric | Pairwise Comparison | Slope Contrast | Adjusted *P* | Effect Size  High | Effect Size Low |
| --- | --- | --- | --- | --- | --- |
| Bray-Curtis | STI – TH | 0.03 | 0.011 | 202% | 51% |
|  | STI – C | 0.02 | 0.014 | 244% | 68% |
|  | C – TH | 0.01 | 0.265 | 19% | 28% |
| Unweighted UniFrac | STI – TH | 1.00 | 0.001 | 272% | 76% |
|  | STI – C | 0.81 | 0.002 | 366% | 67% |
|  | C – TH | 0.19 | 0.251 | 26% | 28% |
| Weighted UniFrac | STI – TH | Interaction not significant | | | |
|  | STI – C |  |  |  |  |
|  | C – TH |  |  |  |  |

Table S3. Pairwise comparisons and relative effect sizes for the significant interaction effect between ∆beak coloration and beta diversity between Northern cardinal microbiome sample timepoints, organized by treatment and beta diversity metric. STI=Simulated Territorial Intrusion, TH=Temporary Hold, and C=Control. Effect Size “Low” and “High” refer to, respectively, the effect size at the lowest and highest end of the spectrum of values for each ∆beak coloration metric.

| Parameter | Beta Diversity Metric | Pairwise Comparison | Slope Contrast | Adjusted *P* | Effect Size  High | Effect Size Low |
| --- | --- | --- | --- | --- | --- | --- |
| Hue | Bray-Curtis | STI – C | -0.02 | 0.015 | 35% | 171% |
|  |  | STI – TH | -0.02 | 0.017 | 48% | 90% |
|  |  | TH – C | <0.01 | 0.949 | 127% | 189% |
|  | Unweighted UniFrac | STI – C | -0.45 | 0.001 | 17% | 424% |
|  |  | STI – TH | -0.44 | <0.001 | 38% | 275% |
|  |  | TH – C | -0.01 | 0.838 | 192% | 154% |
|  | Weighted UniFrac | Interaction not significant | | | | |
| Brightness | Bray-Curtis | STI – C | -0.02 | 0.065 | 34% | 173% |
|  |  | STI – TH | 0.00 | 0.860 | 25% | 10% |
|  |  | TH – C | -0.01 | 0.261 | 14% | 192% |
|  | Unweighted UniFrac | STI – C | -0.33 | <0.001 | 41% | 199% |
|  |  | STI – TH | -0.24 | 0.749 | 51% | 130% |
|  |  | TH – C | -0.09 | 0.813 | 121% | 153% |
|  | Weighted UniFrac | Interaction not significant | | | | |
| Saturation | Bray-Curtis | Interaction not significant | | | | |
|  | Unweighted UniFrac | STI – C | 0.03 | 0.779 | 163% | 26% |
|  |  | STI – TH | 0.00 | 0.779 | 35% | 116% |
|  |  | TH – C | 0.03 | 0.779 | 250% | 36% |
|  | Weighted UniFrac | Interaction not significant | | | | |

Table S4. Pairwise comparisons and relative effect sizes for the significant interaction effect between ∆glucocorticoid response to capture stress and beta diversity between Northern cardinal microbiome sample timepoints, organized by treatment and beta diversity metric. STI=Simulated Territorial Intrusion, TH=Temporary Hold, and C=Control. Glucocorticoid response measured by corticosterone (CORT) concentrations at three timepoints to encapsulate baseline CORT, speed of response, and scope of response. Effect Size “Low” and “High” refer to, respectively, the effect size at the lowest and highest end of the spectrum of values for each CORT metric.

| Parameter | Beta Diversity Metric | Pairwise Comparison | Slope Contrast | Adjusted *P* | Effect Size  High | Effect Size Low |
| --- | --- | --- | --- | --- | --- | --- |
| Baseline CORT | Bray-Curtis | STI – C | -0.03 | 0.003 | 57% | 266% |
|  |  | TH – C | -0.03 | 0.004 | 33% | 251% |
|  |  | STI – TH | -0.01 | 0.648 | 36% | 106% |
|  | Unweighted UniFrac | STI – C | 0.00 | 0.063 | 52% | 236% |
|  |  | TH – C | 0.00 | 0.005 | 34% | 252% |
|  |  | STI – TH | 0.00 | 0.085 | 28% | 6% |
|  | Weighted UniFrac | Interaction not significant | | | | |
| CORT speed | Bray-Curtis | Interaction not significant | | | | |
|  | Unweighted UniFrac | Interaction not significant | | | | |
|  | Weighted UniFrac | Interaction not significant | | | | |
| CORT scope | Bray-Curtis | STI – C | 0.02 | 0.023 | 181% | 34% |
|  |  | TH – C | 0.03 | 0.023 | 251% | 34% |
|  |  | STI – TH | -0.01 | 0.437 | 28% | 0.3% |
|  | Unweighted UniFrac | STI – C | 0.00 | 0.020 | 213% | 38% |
|  |  | TH – C | 0.00 | 0.233 | 208% | 8% |
|  |  | STI – TH | 0.00 | 0.983 | 102% | 32% |
|  | Weighted UniFrac | STI – C | <0.01 | 0.450 | 135% | 12% |
|  |  | TH – C | <0.01 | 0.057 | 343% | 39% |
|  |  | STI – TH | >-0.01 | 0.102 | 60% | 144% |

Table S5. List of Amplicon Sequence Variants in post-treatment cloacal microbiome samples whose relative abundances significantly correlated with ∆Shannon diversity between pre- and post-treatment timepoints. The majority are genera (italicized), while non-italicized Amplicon Sequence Variants represent orders or families.

| Amplicon Sequence Variant | Coefficient ± se | *p* |
| --- | --- | --- |
| *Microvirga* | 0.68 ± 0.18 | 0.001 |
| *67-14* | 0.49 ± 0.12 | <0.001 |
| *Novosphingobium* | 0.79 ± 0.21 | 0.001 |
| *Gordonia* | 1.58 ± 0.46 | 0.001 |
| Microbacteriaceae | 1.48 ± 0.43 | 0.001 |
| *uncultured JG30-KF-CM45* | 0.78 ± 0.26 | 0.005 |
| *uncultured JG30-KF-CM45* | 0.87 ± 0.29 | 0.005 |
| *Nakamurella* | 0.85 ± 0.29 | 0.005 |
| *uncultured Rubrobacteria* | 0.43 ± 0.14 | 0.005 |
| *Paenibacillus* | 0.81 ± 0.27 | 0.005 |
| *Candidatus_Nitrocosmicus* | 0.83 ± 0.27 | 0.003 |
| *Mycobacterium* | 1.18 ± 0.38 | 0.003 |
| *Mycobacterium* | 0.55 ± 0.19 | 0.005 |
| *Novosphingobium* | 0.54 ± 0.18 | 0.004 |
| *Bosea* | 0.74 ± 0.25 | 0.005 |
| *uncultured Gaiellales* | 0.45 ± 0.16 | 0.007 |
| *Nocardioides* | 0.67 ± 0.23 | 0.007 |
| *Mycobacterium* | 1.33 ± 0.46 | 0.006 |
| *Bosea* | 0.99 ± 0.35 | 0.007 |
| *Devosia* | 1.22 ± 0.44 | 0.008 |
| *Bacillus* | 0.29 ± 0.11 | 0.009 |
| *Microvirga* | 0.88 ± 0.32 | 0.009 |
| *Leucobacter* | 0.51 ± 0.20 | 0.013 |
| *Burkholderia-Caballeronia-Paraburkholderia* | 0.59 ± 0.23 | 0.014 |
| *uncultured Beijerinckiaceae* | 0.47 ± 0.18 | 0.013 |
| *Methylobacterium-Methylorubrum* | 1.14 ± 0.44 | 0.014 |
| *67-14* | 0.65 ± 0.26 | 0.015 |
| *Enterococcus* | 1.39 ± 0.55 | 0.016 |
| *uncultured JG30-KF-CM45* | 0.46 ± 0.19 | 0.017 |
| *Gaiella* | 0.29 ± 0.12 | 0.020 |
| *Gaiella* | 0.54 ± 0.22 | 0.020 |
| *Rhodococcus* | 0.71 ± 0.29 | 0.019 |
| Intrasporangiaceae | 0.78 ± 0.32 | 0.018 |
| *Aureimonas* | 0.72 ± 0.30 | 0.019 |
| *Achromobacter* | 0.85 ± 0.34 | 0.018 |
| *Aeromicrobium* | 0.45 ± 0.19 | 0.024 |
| *Raoultibacter* | 0.48 ± 0.20 | 0.024 |
| Solirubrobacterales | 0.58 ± 0.25 | 0.025 |
| *67-14* | 0.57 ± 0.25 | 0.026 |
| Solirubrobacteraceae | 0.57 ± 0.25 | 0.026 |
| *Pseudolabrys* | 0.25 ± 0.10 | 0.023 |
| *Rhodoplanes* | 0.38 ± 0.16 | 0.024 |
| *Reyranella* | 0.38 ± 0.16 | 0.026 |
| *Campylobacter* | -1.33 ± 0.58 | 0.028 |
| *Paracoccus* | 0.66 ± 0.29 | 0.028 |
| *Gordonia* | 0.51 ± 0.23 | 0.030 |

Table S6. Descriptive statistics by group for diversity measures. Treatment group designations: C = Control, STI = Simulated Territorial Intrusion, TH = Temporary Hold. CORT stands for corticosterone. All measures reported are the mean value ± standard error.

| Treatment Group | Shannon Diversity | Faith’s Phylogenetic Diversity | Bray-Curtis | Unweighted UniFrac | Weighted UniFrac |
| --- | --- | --- | --- | --- | --- |
| C | -0.41 ± 0.25 | -0.37 ± 0.32 | 0.08 ± 0.01 | 0.08 ± 0.01 | 0.01 ± 0.001 |
| STI | -0.05 ± 0.34 | 0.04 ± 0.23 | 0.08 ± 0.01 | 0.08 ± 0.01 | 0.01 ± 0.001 |
| TH | 0.16 ± 0.44 | 0.20 ± 0.31 | 0.10 ± 0.01 | 0.11 ± 0.01 | 0.01 ± 0.001 |

Table S7. Descriptive statistics by group for fitness-associated measures. Treatment group designations: C = Control, STI = Simulated Territorial Intrusion, TH = Temporary Hold. CORT stands for corticosterone. All measures reported are the mean value ± standard error for the change in value from pre-treatment to post-treatment sample.

| Treatment Group | Body Condition | Beak Hue | Beak Saturation | Beak Brightness | Baseline CORT | CORT Speed | CORT Scope |
| --- | --- | --- | --- | --- | --- | --- | --- |
| C | -0.03 ± 0.01 | 0.005 ± 0.006 | 0.01 ± 0.02 | -0.004 ± 0.01 | 0.24 ± 2.40 | 0.08 ± 0.10 | 3.28 ± 4.34 |
| STI | -0.03 ± 0.01 | 0.001 ± 0.007 | 0.02 ± 0.03 | -0.001 ± 0.02 | 3.34 ± 1.92 | -0.16 ± 0.15 | 4.64 ± 5.81 |
| TH | -0.004 ± 0.02 | 0.005 ± 0.009 | -0.003 ± 0.02 | -0.01 ± 0.01 | 0.94 ± 2.45 | -0.08 ± 0.10 | -4.34 ± 4.38 |

Table S8. Table of Amplicon Sequence Variants (ASVs) identified as contaminants. Contaminants were identified in R package decontam analyzing frequency and prevalence. For frequency (Freq), decontam uses the frequency of each ASV as a function of the input DNA concentration. For prevalence (Prev), the presence or absence across samples of each ASV in true positive samples is compared to the prevalence in negative controls. An asterisk indicates unknown taxonomy at the given level.

| p | 0.042 | 0.031 | 0.017 | 0.004 | 0.028 | 0.045 | 0.041 | 0.013 | 0.010 | 0.033 | 0.039 | 0.017 | 0.002 | 0.006 | 0.022 | 0.031 | 0.025 |
| --- | --- | --- | --- | --- | --- | --- | --- | --- | --- | --- | --- | --- | --- | --- | --- | --- | --- |
| p Prev | 0.549 | 0.549 | 0.533 | 0.533 | 0.533 | 0.533 | 0.549 | 0.549 | 0.533 | 0.533 | 0.565 | 0.533 | 0.533 | 0.533 | 0.533 | 0.533 | 0.084 |
| p Freq | 0.013 | 0.009 | 0.005 | 0.001 | 0.008 | 0.014 | 0.013 | 0.003 | 0.002 | 0.010 | 0.011 | 0.004 | 0.000 | 0.001 | 0.006 | 0.009 | 0.046 |
| Prev | 3 | 3 | 3 | 2 | 3 | 2 | 3 | 3 | 3 | 2 | 11 | 3 | 2 | 2 | 4 | 2 | 13 |
| Freq | 2.6e-06 | 2.6e-06 | 7.0e-06 | 4.2e-06 | 4.1e-06 | 1.2e-05 | 4.9e-06 | 7.6e-06 | 4.1e-06 | 2.0e-06 | 2.5e-05 | 7.5e-06 | 2.8e-06 | 4.1e-06 | 1.70E-04 | 4.1e-06 | 0.002 |
| EPITHET | * | uncultured bacterium | * | uncultured Acidobacteriales | uncultured bacterium | * | * | metagenome | uncultured actinobacterium | * | * | * | uncultured bacterium | * | * | uncultured bacterium | geothermalis |
| GENUS | Bryobacter | Aurantisolimonas | uncultured | uncultured | uncultured | Gemmataceae | Actinoplanes | PeM15 | Acidothermus | Nocardioides | Gaiella | IMCC26256 | MB-A2-108 | Chryseobacterium | Lachnoclostridium | uncultured | Deinococcus |
| FAMILY | Bryobacteraceae | Chitinophagaceae | uncultured | uncultured | Pirellulaceae | Gemmataceae | Micromonosporaceae | PeM15 | Acidothermaceae | Nocardioidaceae | Gaiellaceae | IMCC26256 | MB-A2-108 | Weeksellaceae | Lachnospiraceae | Chroococcidiopsaceae | Deinococcaceae |

| p | 0.030 | 0.006 | 0.032 | 0.032 | 0.005 | 0.016 | 0.032 | 0.022 | 0.048 | 0.050 | 0.009 | 0.030 | 0.007 | 0.028 | 0.032 | 0.005 |
| --- | --- | --- | --- | --- | --- | --- | --- | --- | --- | --- | --- | --- | --- | --- | --- | --- |
| p Prev | 0.533 | 0.533 | 0.020 | 0.051 | 0.007 | 0.004 | 0.029 | 0.533 | 0.533 | 0.533 | 0.533 | 0.084 | 0.013 | 0.051 | 0.051 | 0.007 |
| p Freq | 0.009 | 0.001 | 0.252 | 0.100 | 0.097 | 0.582 | 0.176 | 0.006 | 0.015 | 0.016 | 0.002 | 0.057 | 0.064 | 0.084 | 0.100 | 0.097 |
| Prev | 2 | 5 | 14 | 3 | 63 | 6 | 15 | 2 | 5 | 4 | 5 | 11 | 19 | 14 | 3 | 63 |
| Freq | 2.6e-06 | 7.7e-06 | 0.002 | 1.9e-05 | 0.004 | 0.001 | 0.001 | 6.0e-06 | 7.2e-06 | 8.9e-06 | 1.7e-05 | 2.3e-05 | 0.001 | 0.0003 | 1.9e-05 | 0.004 |
| EPITHET | Solirubrobacterales bacterium | alginolyticus | * | * | * | * | * | * | * | * | * | * | * | diminuta | * | * |
| GENUS | 67-14 | Paenibacillus | Staphylococcus | Geobacillus | Geobacillus | Anaerococcus | Phyllobacterium | Sphingomonas | Novosphingobium | Candidatus | Allorhizobium-Neorhizobium-Pararhizobium-Rhizobium | Ramlibacter | * | Brevundimonas | Geobacillus | Geobacillus |
| FAMILY | 67-14 | Paenibacillaceae | Staphylococcaceae | Bacillaceae | Bacillaceae | Peptostreptococcales-Tissierellales | Rhizobiaceae | Sphingomonadaceae | Sphingomonadaceae | Geminicoccaceae | Rhizobiaceae | Comamonadaceae | Comamonadaceae | Caulobacteraceae | Bacillaceae | Bacillaceae |

Table S9. The cross reactivities are listed below for the Arbor Assays Enzyme Immunoassay kit (Catalog No. K014-H) used to estimate corticosterone from blood samples collected from male Northern cardinals.

| Steroid | Cross Reactivity (%) |
| --- | --- |
| Corticosterone | 100% |
| 1-dehydrocorticosterone | 18.9% |
| Desoxycorticosterone | 12.3% |
| 1α-hydroxycorticosterone | 3.3% |
| 11-dehydrocorticosterone | 2.4% |
| Tetrahydrocorticosterone | 0.76% |
| Aldosterone | 0.62% |
| Cortisol | 0.38% |
| Progesterone | 0.24% |
| Dexamethasone | 0.12% |
| Testostrone | 0.03% |
| Corticosterone-21-hemisuccinate | < 0.1% |
| Cortisone | < 0.08% |
| Estradiol | < 0.08% |
| 17-hydroxyprogesterone | < 0.01% |
| Allopregnanolone | < 0.01% |
| Dehydroepiandrosterone sulfate | < 0.01% |
| Estrone-3-glucuronide | < 0.01% |
| Estrone-3-sulfate | < 0.01% |

FIGURES

A. B.


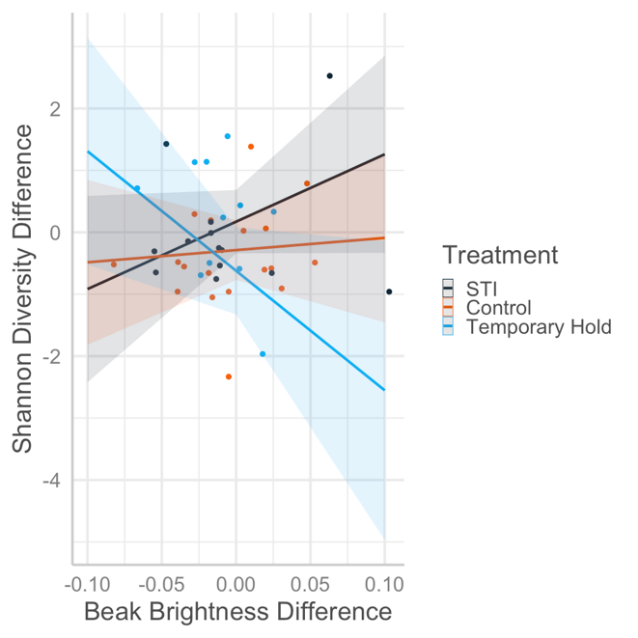

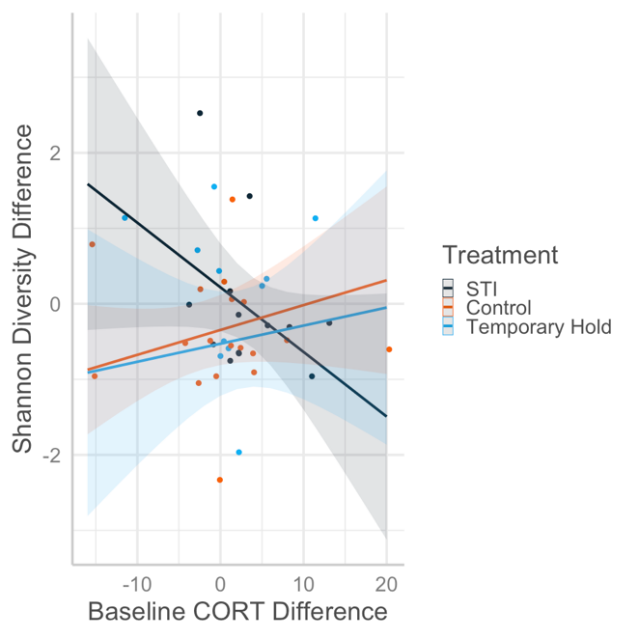


C.


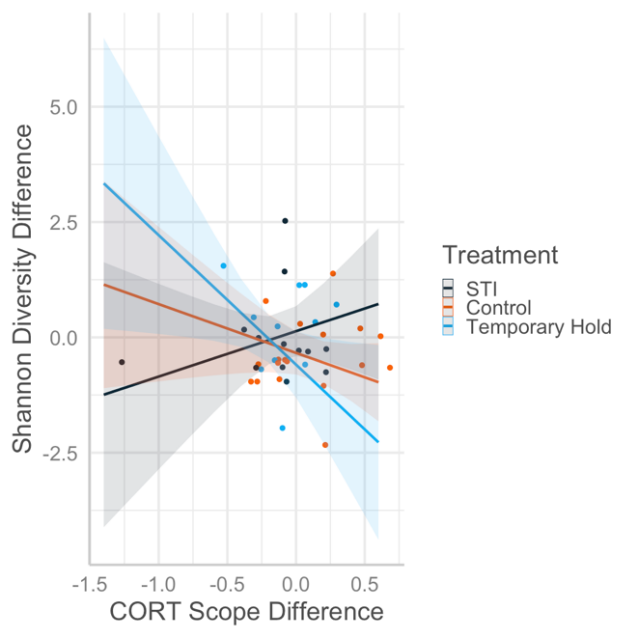


Figure S1. ∆Shannon Diversity between pre- and post-treatment cloacal microbiome samples of Northern cardinal males. Interaction effects were seen between treatment and change in A) beak brightness (p=0.060), B) baseline corticosterone (CORT; p=0.099), and C) CORT speed (p=0.086), but none of these were significant after multiple test correction. “STI” stands for simulated territorial intrusion.

1. B.


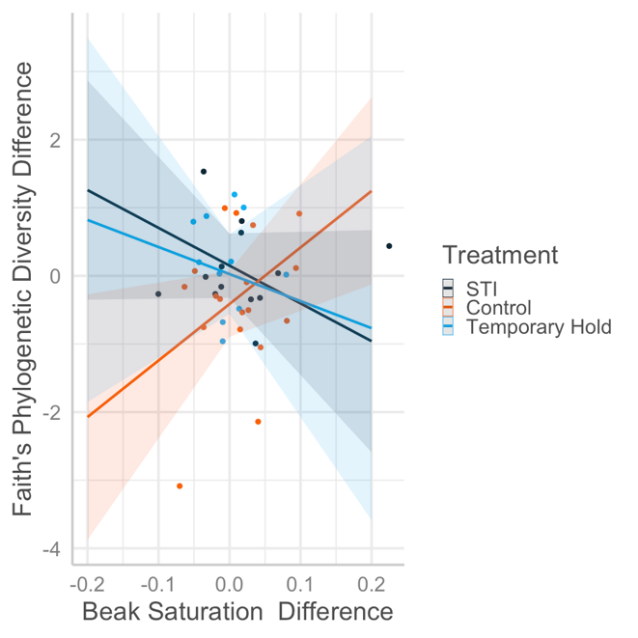

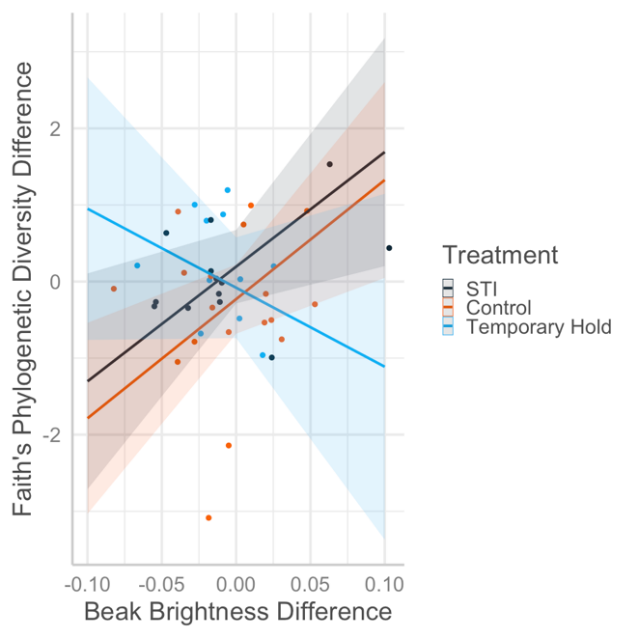


Figure S2. Change in Faith’s Phylogenetic Diversity between pre- and post-treatment cloacal microbiome samples of Northern cardinal males. Significant interaction effects were seen between treatment and change in A) beak saturation (p=0.034), and B) beak brightness (p=0.058), but none of these were significant after multiple test correction. “STI” stands for simulated territorial intrusion.

A. B.


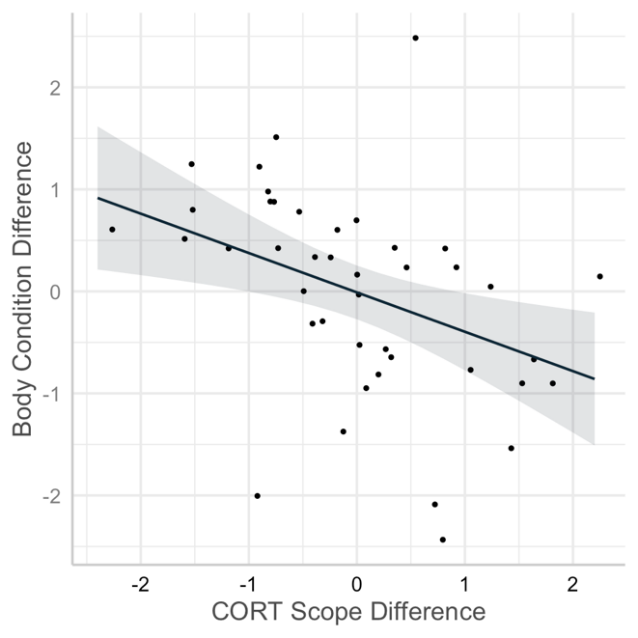

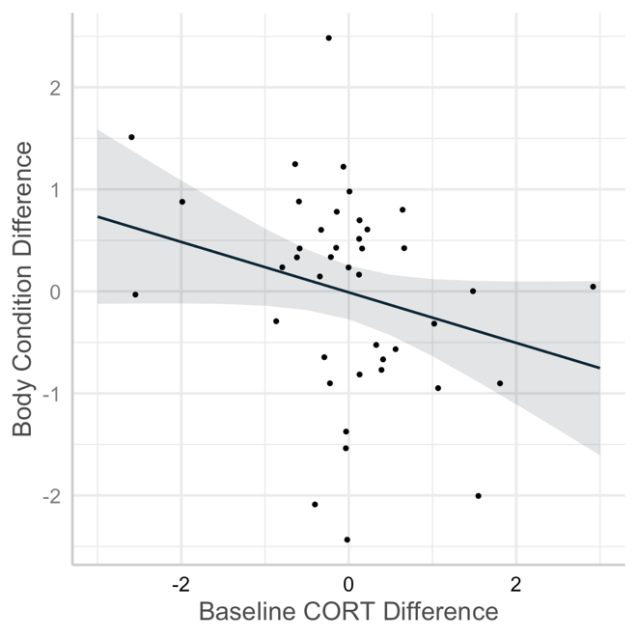


Figure S3. Data and trendlines from a post-hoc generalized linear model of samples collected from male Northern cardinals documenting changes between capture timepoints in body condition and corticosterone (CORT) response to stress (model: “change in body condition” ~ “change in baseline CORT” + “change in CORT scope”). Specifically, A) a significant relationship between the changes in CORT scope and body condition, and B) a relationship approaching significance between changes in baseline corticosterone and body condition.


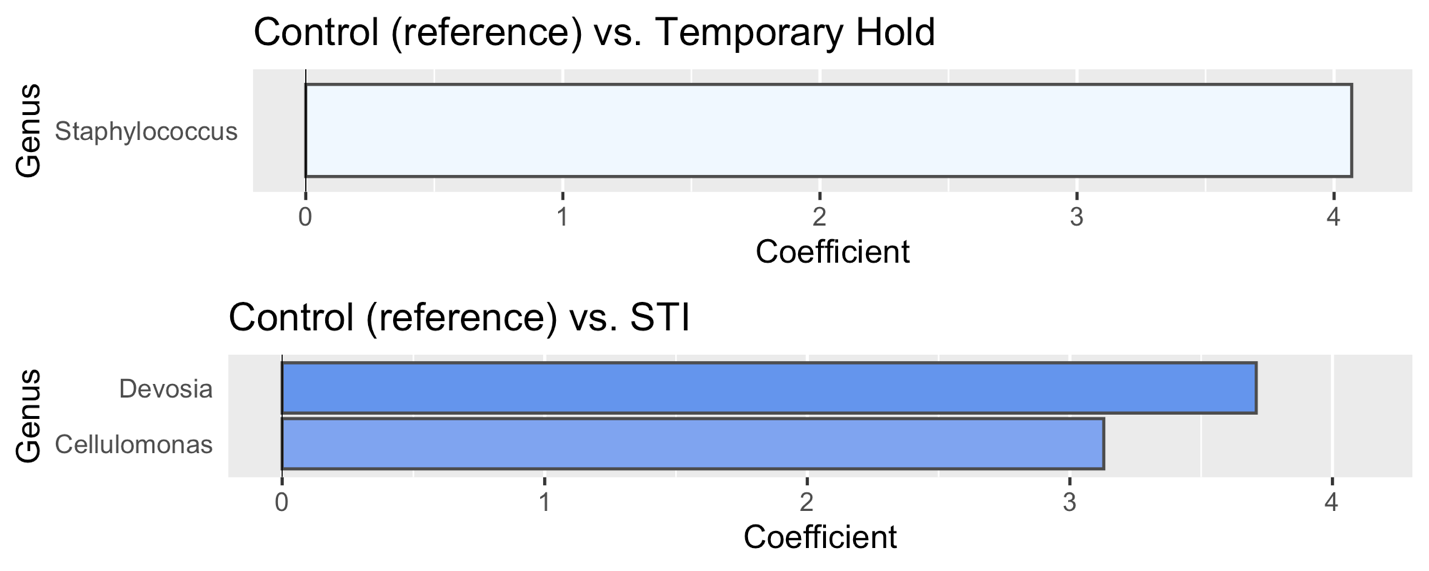


Figure S4. Divergence barplots showing the coefficients from differential abundance modeling. Model coefficients in the top panel compare abundances of Amplicon Sequence Variants that were significantly differentially abundant in post-treatment cloacal microbiome samples of male Northern cardinals receiving various treatments between 2 sample timepoints. The top plot shows birds in the Temporary Hold group compared to the Control group, while the bottom panel compares that of the Simulated Territorial Intrusion (STI) group relative to the Control group.


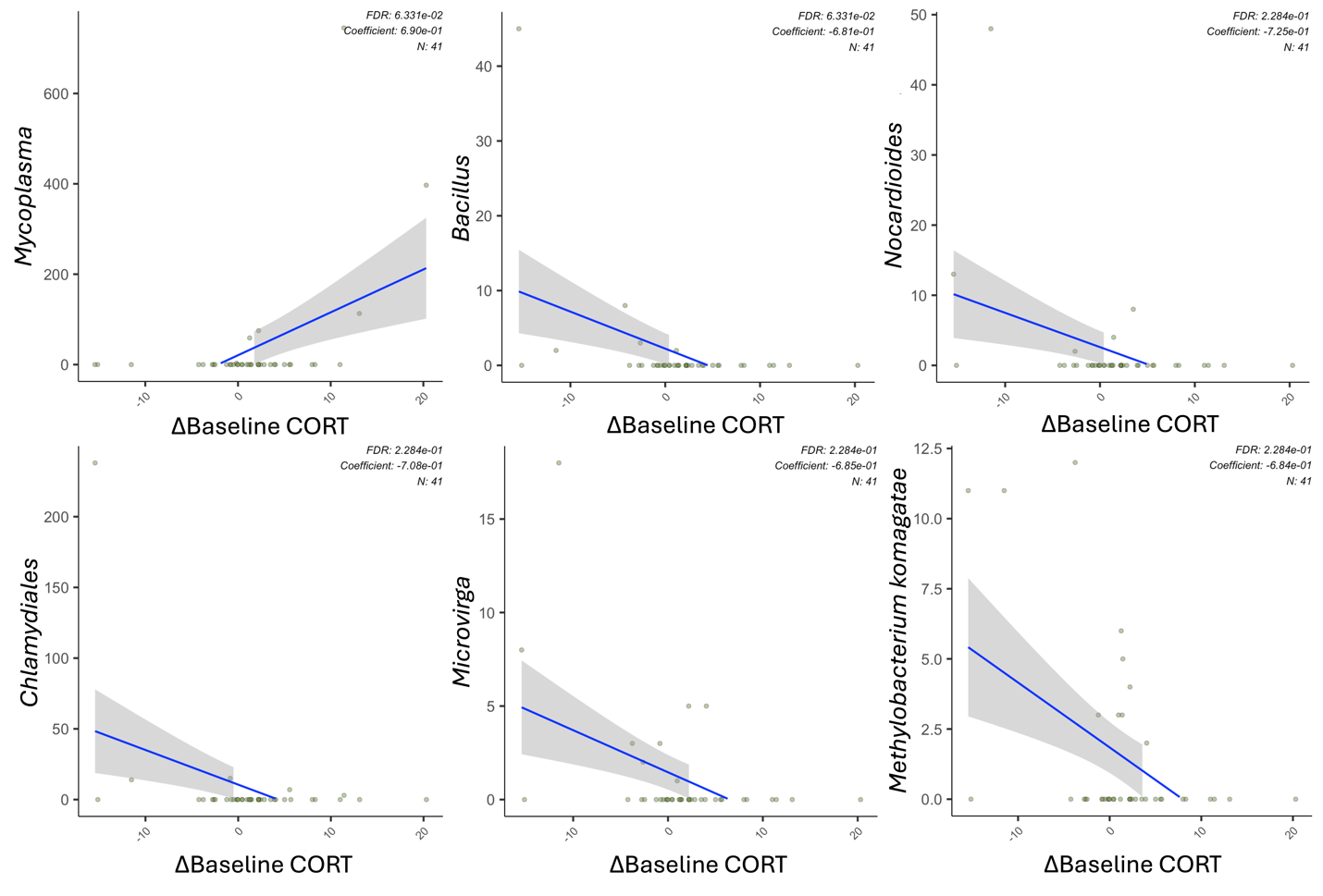


Figure S5. Scatterplots showing significant correlations between change (∆) in baseline corticosterone (CORT) concentration and relative abundances of six Amplicon Sequence Variants found in post-treatment cloacal microbiome samples of male Northern cardinals.
